# Initial experience in implementing quantitative DCE-MRI to predict breast cancer therapy response in a multi-center and multi-vendor platform setting

**DOI:** 10.3389/fonc.2024.1395502

**Published:** 2024-11-29

**Authors:** Brendan Moloney, Xin Li, Michael Hirano, Assim Saad Eddin, Jeong Youn Lim, Debosmita Biswas, Anum S. Kazerouni, Alina Tudorica, Isabella Li, Mary Lynn Bryant, Courtney Wille, Chelsea Pyle, Habib Rahbar, Su Kim Hsieh, Travis L. Rice-Stitt, Suzanne M. Dintzis, Amani Bashir, Evthokia Hobbs, Alexandra Zimmer, Jennifer M. Specht, Sneha Phadke, Nicole Fleege, James H. Holmes, Savannah C. Partridge, Wei Huang

**Affiliations:** ^1^ Advanced Imaging Research Center, Oregon Health and Science University, Portland, OR, United States; ^2^ Department of Radiology, University of Washington, Seattle, WA, United States; ^3^ Department of Radiology, University of Iowa, Iowa City, IA, United States; ^4^ Biostatistics Shared Resource, Knight Cancer Institute, Oregon Health and Science University, Portland, OR, United States; ^5^ Department of Diagnostic Radiology, Oregon Health and Science University, Portland, OR, United States; ^6^ Institute for Clinical and Translational Science, University of Iowa, Iowa City, IA, United States; ^7^ Fred Hutchinson Cancer Center, Seattle, WA, United States; ^8^ Department of Pathology, Oregon Health and Science University, Portland, OR, United States; ^9^ Department of Pathology, University of Washington, Seattle, WA, United States; ^10^ Holden Comprehensive Cancer Center, University of Iowa, Iowa City, IA, United States; ^11^ Department of Pathology, University of Iowa Hospitals and Clinics, Iowa City, IA, United States; ^12^ Hematology and Medical Oncology Division, Knight Cancer Institute, Oregon Health and Science University, Portland, OR, United States; ^13^ Division of Hematology and Oncology, University of Washington, Seattle, WA, United States; ^14^ Department of Internal Medicine, University of Iowa Hospitals and Clinics, Iowa City, IA, United States

**Keywords:** breast cancer, therapy response, dynamic contrast-enhanced (DCE) MRI, pharmacokinetics, K^trans^, water exchange, multi-center, multi-vendor platform

## Abstract

Quantitative dynamic contrast-enhanced (DCE) MRI as a promising method for the prediction of breast cancer response to neoadjuvant chemotherapy (NAC) has been demonstrated mostly in single-center and single-vendor platform studies. This preliminary study reports the initial experience in implementing quantitative breast DCE-MRI in multi-center (MC) and multi-vendor platform (MP) settings to predict NAC response. MRI data, including B_1_ mapping, variable flip angle (VFA) measurements of native tissue R_1_ (R_1,0_), and DCE-MRI, were acquired during NAC at three sites using 3T systems with Siemens, Philips, and GE platforms, respectively. High spatiotemporal resolution DCE-MRI was performed using similar vendor product sequences with k-space undersampling during acquisition and view sharing during reconstruction. A breast phantom was used for quality assurance/quality control (QA/QC) across sites. The Tofts model (TM) and shutter-speed model (SSM) were used for pharmacokinetic (PK) analysis of the DCE data. Additionally, tumor region of interest (ROI)- *vs*. voxel-based analyses in combination with the use of VFA-measured R_1,0_ *vs*. fixed, literature-reported R_1,0_ were investigated to determine the optimal analysis approach. Results from 15 patients who completed the study are reported. Voxel-based PK analysis using fixed R_1,0_ was deemed the optimal approach, which allowed the inclusion of data from one vendor platform where VFA measurements produced ≥100% overestimation of R_1,0_. The semi-quantitative signal enhancement ratio (SER) and quantitative PK parameters outperformed the tumor longest diameter (LD) in the prediction of pathologic complete response (pCR) *vs.* non-pCR after the first NAC cycle, whereas K^trans^ consistently provided more accurate predictions than both SER and LD after the first NAC cycle and at the NAC midpoint. Both TM and SSM K^trans^ and k_ep_ were excellent predictors of response at the NAC midpoint with ROC AUC >0.90, while the SSM parameters (AUC ≥0.80) performed better than their TM counterparts (AUC <0.80) after the first NAC cycle. The initial experience of this ongoing study indicates the importance of QA/QC using a phantom and suggests that deploying voxel-based PK analysis using a fixed R_1,0_ may mitigate random errors from R_1,0_ measurements across platforms and potentially eliminate the need for B_1_ and VFA acquisitions in MC and MP trials.

## Introduction

Neoadjuvant chemotherapy (NAC) is frequently used in the standard of care (SoC) to treat patients with locally advanced breast cancer to downstage the disease and facilitate breast-conserving surgery. In addition, the setting of NAC systemic treatment affords the opportunity to assess the pathologic response to NAC. Studies have shown that pathologic complete response (pCR) or minimal residual disease following NAC is prognostic for survival (1–6). However, since assessment of pathologic response can only be ascertained from surgical tumor specimens after NAC has already been completed, the treating clinician’s options to tailor therapy regimens during NAC to improve pathologic response outcome and consequently survival are limited. Therefore, minimally invasive methods that can provide an accurate prediction of response in the early stages of NAC are urgently needed. With many innovative treatment regimens using targeted therapies and/or immunotherapies being tested in clinical trials for breast cancer treatment, improved capability of accurate and early prediction of pathologic response to NAC may allow rapid individualized regimen de-escalation/alteration for responding/non-responding breast cancer patients in the future, facilitating precision medicine and leading to improved treatment outcomes.

In current SoC and clinical trials, the measurement of imaging tumor size change according to the Response Evaluation Criteria in Solid Tumors (RECIST) guidelines (7, 8) is the standard approach to assess tumor response to therapy. However, many studies have shown that tumor size changes in response to therapy, especially targeted therapies, often lag well behind changes in the underlying tumor biological functions (9–11), such as perfusion/permeability, cellularity, and metabolism. Therefore, the anatomic imaging approach of tumor size measurement is generally less effective for the early prediction of therapeutic response compared to functional imaging methods. As a noninvasive method for evaluation of microvascular perfusion and permeability, dynamic contrast-enhanced (DCE) MRI has been increasingly used in research settings, including early phase clinical trials, to evaluate breast cancer responses to NAC. There are usually three approaches in the analysis of DCE-MRI time-course data: qualitative curve shape description, calculation of semi-quantitative metrics such as contrast agent (CA) uptake and wash-out slopes, and quantitative pharmacokinetic (PK) modeling to extract parameters, such as K^trans^ (CA volume transfer rate constant) and v_e_ (extravascular, extracellular volume fraction), which are more directly reflective of the underlying biological functions and are in principle independent of scanner vendor platforms and data acquisition details. Thus, compared with qualitative and semi-quantitative DCE-MRI, quantitative DCE-MRI is hypothetically a more desirable approach for the assessment of cancer therapy response. Using summary receiver operating characteristic (SROC) analysis, a recent meta-analysis (12) of 14 published studies including 739 patients showed that the area under the curve (AUC), sensitivity, and specificity of K^trans^ for early discrimination of pCR and non-pCR after one to two NAC cycles were 0.90, 84%, and 83%, respectively. However, the promise of K^trans^ as an imaging biomarker for the prediction of breast cancer response to NAC has been demonstrated mostly by single-site and single-vendor platform DCE-MRI studies (13) that often sacrificed spatial resolution and/or coverage for the high temporal resolution necessary for PK modeling of the time-course data. There remain significant technical challenges in implementing quantitative DCE-MRI in multi-center (MC) and multi-vendor platform (MP) settings to assess breast cancer response to NAC, as many technical aspects in data acquisition and analysis, from temporal resolution to selection of the PK model and software tool, can affect the accuracy and precision of the derived PK parameters (14–17). Therefore, it is of paramount importance to standardize data acquisition and analysis strategies in MC and MP study settings (14, 15).

In this study, through preliminary analysis of data from an ongoing project, we report our initial experience in implementing quantitative DCE-MRI with simultaneous high spatial and temporal resolutions in an MC and MP setting to predict breast cancer response to NAC, compare predictive performances among quantitative and semi-quantitative DCE-MRI parameters, and tumor size measurement, and make initial best-practice recommendations with regard to PK analysis of breast DCE-MRI data acquired from different sites with different vendor platforms.

## Methods

### Study schema and patient cohort

With local internal review board (IRB) approval, breast cancer patients treated with SoC NAC were enrolled with informed consent at three sites—Oregon Health & Science University (OHSU), University of Washington (UW), and University of Iowa (UI)—to participate in a longitudinal research MRI study. Four MRI sessions were performed before, during, and after the NAC course: Visit 1 (V1, before NAC), Visit 2 (V2, after the first cycle of NAC), Visit 3 (V3, at the midpoint of NAC; generally, after the completion of the first drug regimen but before the start of the second drug regimen), and Visit 4 (V4, after NAC but before surgery). Each patient’s pCR (defined as no residual invasive disease in the breast or axilla) or non-pCR status after NAC was determined by pathological analysis of the surgical tumor specimens as per the SoC procedures.

Fifteen patients across the three sites completed NAC treatment and MRI studies with pathologic response outcomes to date. **Table 1** lists the clinicopathological characteristics of these 15 patients, with six of them achieving pCR (40%). The imaging results and correlations with the pathological response outcomes are reported below.

**Table 1:** Clinicopathological characteristics of the patient cohort (N = 15).

Age (mean ± SD)	50.4 ± 11.2 years
Tumor type	
IDC	13
ILC	2
Tumor grade	
III	9
II	6
Pre-NAC tumor LD (mean ± SD)	37.8 ± 18.2 mm
Breast cancer subtypes	
HR (ER or PR) +	8
HER2 +	4
TN	5
Pathologic response to NAC	
pCR	6
non-pCR	9
RCB Class I	4
RCB Class II	3
RCB Class III	2

SD, standard deviation; IDC, invasive ductal carcinoma; ILC, invasive lobular carcinoma; NAC, neoadjuvant chemotherapy; LD, longest diameter; HR, hormonal receptor; ER, estrogen receptor; PR, progesterone receptor; HER2, human epidermal growth factor receptor 2; TN, triple negative; RCB, residual cancer burden; pCR, pathologic complete response.

### MRI data acquisition

A 3T MRI scanner was used at each site for MRI data acquisition with each site employing a unique vendor platform, including Siemens (Siemens Healthineers, Erlangen, Germany), General Electric (GE Healthcare, Waukesha, WI), and Philips (Philips Healthcare, Best, the Netherlands). The vendor platform and software versions are listed in **Table 2**. Each MRI session consisted of a scout scan and the following scans in the axial plane with bilateral full breast coverage: T_2_-weighted MRI with fat suppression, T_1_-weighted MRI without fat suppression, axial diffusion-weighted MRI (DWI), B_1_ mapping, variable flip angle (VFA) gradient-echo (GRE) MRI for mapping of native tissue T_1_ (T_1,0_) (18), and DCE-MRI. Since this study mainly reports the results of DCE-MRI for the prediction of breast cancer response to NAC, only the sequences and acquisition parameters relevant to DCE-MRI quantification on the three vendor platforms are summarized in **Table 2**.

Table 2MRI data acquisition details for the three vendor platforms (Siemens, Philips, and GE).

**Table:** 

A. Platform and Hardware				
vendor	field strength & platform	Software version	RF coil transmit	RF coil receiver
GE	3T Signa Premier	RX29.1_R04_2313.a	built-in T/R body coil	Sentinelle bilateral 16 channel breast coil
Philips	3T Achieva/Ingenia	5.7.1sp2/sp3	Q-body	Mammotrak/dStream 16 channel breast coil
Siemens	3T Prisma	Syngo VE11C/XA30	built-in T/R body coil	Sentinelle bilateral 16 channel breast coil

**Table:** 

B. Relevant Pulse Sequence Details								
		sequence name	TR (ms)	TE (ms)	FA (°)	reconstructed slice thickness (mm)	number of slices	in-plane matrix size as acquired
B_1_	GE	Block-Siegert 2D B1map	19	6.3	23	10	18	64 × 64
	Philips	3D FFE	30	2.3	60	6	28	90 × 96
	Siemens	2D TFL	9,280	2.0	8	5	34	64 × 64
VFA	GE	3D GRE*	5.5	1.008	3, 9, 15	1.4	130	320 × 320
	Philips	3D GRE*	5.6	2.8	3,9,15	1	170	240 × 360
	Siemens	3D GRE*	10	2.8	3,9,15	1.4	112–128	160 × 160
DCE	GE	DISCO	5	0.944	10	1.4	130	320 × 320
	Philips	4D TRAK XD	5.9	2.8	10	1.5	113	240 × 360
	Siemens	TWIST 3D	6.2	2.9	10	1.4	112–128	320 × 320

**Table:** 

B. Relevant Pulse Sequence Details (Continued)						
		sequence name	in-plane matrix size reconstructed	in-plane field of view (cm)	temporal resolution (s)	acquisition time(min)
B_1_	GE	Block-Siegert 2D B1map	64 × 64	34 × 34	NA	0:40
	Philips	3D FFE	144 × 144	24 × 36	NA	3:47
	Siemens	2D TFL	64 × 64	32 × 32	NA	0:19
VFA	GE	3D GRE*	512 × 512	34 × 34	NA	1:26
	Philips	3D GRE*	480 × 480	24 × 36	NA	1:15
	Siemens	3D GRE*	320 × 320	32 × 32 or 34 × 34	NA	2:12
DCE	GE	DISCO	512 × 512	34 × 34	15	8:30–9:30
	Philips	4D TRAK XD	528 × 528	24 × 36	15	9:29
	Siemens	TWIST 3D	320 × 320	32 × 32 or 34 × 34	12–16	9:00–9:30

**Table:** 

B. Relevant Pulse Sequence Details (continued)							
		sequence name	fat saturation method	parallel imaging method	parallel imaging acceleration factor	receiver bandwidth(Hz/pix)	number of frames
B_1_	GE	Block-Siegert 2D B1map	NA	NA	NA	488.4	1
	Philips	3D FFE	NA	NA	NA	499	1
	Siemens	2D TFL	NA	NA	NA	490	1
VFA	GE	3D GRE*	ASPIR/SPAIR	ARC	3	781.25	1
	Philips	3D GRE*	Water selective excitation with binomial pulses (proset)	SENSE	2	947	1
	Siemens	3D GRE*	Water excitation	GRAPPA	2	490	1
DCE	GE	DISCO	ASPIR/SPAIR	ARC	3	781.25	32
	Philips	4D TRAK XD	proset	SENSE	2.5 (P reduction RL), 1.1 (S reduction FH)	947	34
	Siemens	TWIST 3D	Water excitation	GRAPPA	3	630	30–36

FFE, fast field echo; SENSE, SENSitivity Encoding; TFL, turbo-Flash; FL, Flash; GRE, gradient echo; ARC, Autocalibrating Reconstruction for Cartesian imaging; GRAPPA, GeneRalized Autocalibrating Partially Parallel Acquisition; ASPIR/SPAIR, Adiabatic Spectral Inversion Recovery/Spectral Attenuated Inversion Recovery; DISCO, DIfferential Subsampling with Cartesian Ordering; 4D TRAK, 4D Time-Resolved Angiography using Keyhole; TWIST, Time-resolved angiography WIth Stochastic Trajectories. *: The same k-space-undersampling and view-sharing sequence used for DCE acquisition was used for VFA acquisition on each vendor platform. However, when used for a single-time-point acquisition like VFA, these sequences are equivalent to a conventional GRE sequence without performing k-space-undersampling and view-sharing.

To achieve simultaneous high spatial and high temporal resolution DCE-MRI, similar product 3D GRE-based sequences employing Cartesian k-space undersampling in acquisition and view-sharing in reconstruction were used at the three sites for DCE-MRI data acquisition: Time-resolved angiography WIth Stochastic Trajectories (TWIST) (19–21), 4D Time-Resolved Angiography using keyhole (4D-TRAK) (22), and DIfferential Subsampling with Cartesian Ordering (DISCO) (23, 24) on Siemens, Philips, and GE platforms, respectively. Except for the first DCE time frame where full k-space data were acquired, the k-space data acquired for each remaining frame included the center region of the k-space and a portion of the peripheral k-space. For each of the three vendor sequences, the center region of the k-space was set at 15% of the full k-space, and the peripheral portion was set at 20% of the peripheral k-space. For VFA acquisitions, full k-space GRE MRI data were acquired, and three FAs of 3°, 9°, and 15° were used with the estimated Ernst angle positioned between the largest and smallest angles. The selection of these three FAs was automatically determined by a product T_1_ mapping sequence from one vendor following the entry of literature breast tumor R_1_ (= 1/T_1_) value of approximately 0.6 s^−1^ at 3T (25–27). B_1_ mapping, VFA-MRI, and DCE-MRI were spatially aligned during postprocessing.

For DCE-MRI, the same gadolinium-based CA, Prohance (Bracco Diagnostics Inc., Township, NJ, USA), dose (0.1 mmol/kg), injection rate (2 mL/s using a programmable power injector), and injection site (antecubital vein) were used at all three institutions. Intravenous administration of CA was initiated at the beginning of the third DCE frame acquisition, followed by a 20-mL saline flush at the same injection rate.

For quality assurance and quality control (QA/QC) of this MC and MP study, a bilateral breast phantom with a diffusion side and T_1_ side (CaliberMRI, Boulder, CO, USA; https://qmri.com/product/premium-single-wall-breast/) was scanned monthly at the three sites with the same DWI, B_1_ mapping, and VFA-MRI protocols used for the patient study. Data from the T_1_ side with compartments containing breast fibroglandular tissue- and adipose tissue-mimicking materials were used for the QA/QC of quantitative DCE-MRI. B_1_ maps and VFA data from the phantom T_1_ side were used to generate B_1_-corrected R_1_ maps, which were compared with the known ground-truth R_1_ values of the phantom at the experimental temperature.

### MRI data analysis

#### Tumor size measurement and region of interest

Breast tumor longest diameter (LD) was measured by a site radiologist from post-contrast DCE-MRI images according to the RECIST 1.1 guidelines (8) for cases of a single primary tumor or the presence of multiple tumors in the same breast. Under the supervision of the site radiologist, tumor regions of interest (ROIs) were manually drawn by the site investigators on each image slice containing the contrast-enhanced tumor. If multiple tumors were present, ROIs were drawn independently for each tumor.

Together with the ROIs, de-identified B_1_ mapping, VFA-MRI, and DCE-MRI data from the patients enrolled at the two sites (UW and UI), as well as from the phantoms (not de-identified), were submitted to a secure server at the management site (OHSU) for further centralized analysis.

#### Semi-quantitative analysis of DCE-MRI data

In a large multicenter clinical trial (28, 29), functional breast tumor volume calculated based on a semi-quantitative DCE-MRI parameter, the signal enhancement ratio (SER), was shown to be a promising imaging biomarker for predicting breast cancer response to NAC and survival. Following a similar approach, voxel-based SER values within the tumor ROIs were derived from the DCE time-course data using the following equation:


(1) $$\text{SER}=({\text{S}}_{9}-{\text{S}}_{2})/({\text{S}}_{26}-{\text{S}}_{2})$$

where S_2_ is the signal intensity from the second DCE frame, a pre-contrast baseline frame, and S_9_ and S_26_ are the post-contrast signal intensities from the 9th (early phase, approximately 110 s after contrast injection) and 26th (delayed phase, approximately 380 s after contrast injection) DCE frames, respectively.

#### Quantitative PK analysis of DCE-MRI data

##### R_1,0_ calculation with B_1_ correction

To estimate the intrinsic tissue R_1_ (R_1,0_) value before CA arrival for each voxel, a B_1_-corrected R_1_ map was computed from the VFA data for both the phantom and de-identified patient data. Briefly, B_1_ DICOM images were first converted to a B_1_ ratio map based on vendor-provided formalisms. Therefore, voxel-based ratio value quantifies the fractional FA deviation from the nominal input value prescribed in the sequence. For example, a value of 1.0 reflects perfect agreement between the actual and prescribed FA values and 1.2 reflects an FA that is 20% larger than the nominal FA prescribed in the sequence. Each B_1_ acquisition had one or more accompanying image sets (used to calculate B_1_) which provided more anatomical details than the actual B_1_ map; therefore, we used these to co-register the B_1_ maps to the VFA images with the most similar contrast using publicly available ANTs software (30). B_1_ maps were interpolated to allow voxel-wise FA correction in the VFA data. When fitting an R_1_ value for each voxel against the VFA data, B_1_-corrected FA was used instead of the nominal value. Voxel-by-voxel fitting was performed using the standard nonlinear fitting approach using (Equation 2),


(2) $$S\left(\alpha \right)=S_{0}\frac{\left(1-e^{-\left(TR\cdot R_{1}\right)}\right)\text{sin}\left(\alpha \right)}{1-\text{cos}\left(\alpha \right)e^{-\left(TR\cdot R_{1}\right)}}$$

where α is the B_1_-corrected FA that acts as the independent variable in the VFA R_1_ fitting and TR is the repetition time. R_1_ and S_0_ in (Equation 2) are the fitting parameters. The R_1_ in Equation 2 becomes R_1,0_ when the patient VFA data collected before DCE-MRI are fitted to the equation. In addition, it has been shown that potentially different scaling factors may be applied to image intensities across the three different FAs in VFA acquisition on a particular vendor platform (31). Before quantifying phantom R_1_ or tumor R_1,0_ from the VFA data obtained from that platform, corrections of signal intensities were made if differences in scaling factors were observed in the DICOM tags (31).

##### PK analysis of patient DCE-MRI data

A generalized MRI modeling fitting package written in Python was developed at OHSU’s Advanced Imaging Research Center (AIRC). This package includes several DCE PK models. Using this package and several other software tools, a processing workflow was developed specifically for for this MC and MP study. Although the goal of the near future is to make the software package available to all three sites in this study (and eventually to the broader research community) for localized data processing, centralized data processing was used for this initial effort. The PK models used in data analysis are the fast-exchange-limit (FXL) Tofts model (TM) (32, 33) and the simplest fast-exchange-regime (FXR) exchange-sensitized shutter-speed models (SSM) (34, 35). The CA volume transfer rate constant, K^trans^, and extravascular extracellular space (EES) volume fraction, v_e_, were modeled using the TM. For SSM, the unidirectional cellular water efflux rate constant, k_io_, is modeled in addition to K^trans^ and v_e_. The rate constant k_ep_ was calculated as K^trans^/v_e_ in both models. The population-averaged arterial input function (AIF) (36) measured from an axillary artery in a previous single-breast DCE study in the sagittal plane was adopted for all PK modeling in this study. The PK modeling details are provided in the **Supplementary Material**.

##### ROI- and voxel-based PK analysis with measured and fixed R_1,0_

In addition to the use of TM and SSM for PK data analysis, some additional practical considerations in PK modeling were also investigated. These included ROI- *vs.* voxel-based PK analysis in combination with the use of fixed, literature-reported R_1,0_ (*f*R_1,0_ = 0.60 s^−1^) (25–27) *vs.* VFA-measured R_1,0_ (*m*R_1,0_) for four analysis conditions for each PK model: ROI_ *f*R_1,0_, ROI_*m*R_1,0_, voxel_ *f*R_1,0_, and voxel_ *m*R_1,0_. When *m*R_1,0_ is used, the VFA series with FA (= 9°) closest to that of the DCE were used to coregister the measured R_1,0_ maps to DCE baseline data. For ROI-based analysis, multi-slice tumor ROIs were concatenated to form a 3D tumor ROI with a single averaged DCE time course for PK modeling. For voxel-based (within ROIs) analysis, each voxel DCE time course underwent PK modeling. The ROI-based analysis has the advantage of significantly increasing the signal-to-noise ratio (SNR) of the time-course data and much less computing time required for PK modeling, whereas the use of *f*R_1,0_ reduces the imaging time for data acquisition and simplifies data post-processing. K^trans^ has been shown by many research studies to be the best quantitative DCE-MRI biomarker for prediction of breast cancer response to NAC (13). For this MC and MP study, K^trans^ was used as the reference biomarker to investigate the effects of these four different quantitative DCE-MRI analysis approaches using either TM or SSM for PK modeling.

##### Bolus arrival time

The bolus arrival time (BAT) is defined as the delay in the arrival of the CA in the tissue of interest from the artery where the AIF is measured. In general, it is assumed that the CA concentration time-course in the blood plasma (or AIF), C_p_(t), is temporally aligned to match the CA concentration time-course in the tissue, C_t_(t). Typically, this is performed manually or by convention (e.g., based on when the injection occurs), and a single global alignment is chosen. However, the time at which the CA bolus arrives at any given voxel is different owing to differing blood transit times. Even in studies where a high-quality AIF can be measured directly from some arterial voxels visible in the DCE acquisition, it is likely that the AIF needs to be time-shifted for accurate PK analysis of the tissue time-course data of any given voxel. Misalignment can cause biases in all estimated PK model parameters. To reduce these biases (and reduce manual work in time-shifting AIF) we adopted a model-based approach to align the AIF for each tissue voxel curve. To achieve this, we fit a linearized version of TM (37), wrapped in a nonlinear optimizer that solves for a single parameter: BAT. Because this nonlinear problem can have multiple local minima, we performed the optimization in two phases, starting with a brute force search using a coarse grid, followed by an iterative solver (using Powell’s method) for fine-tuning.

##### k_io_ filtering

The sensitivity of DCE-MRI data to water exchange depends on many factors such as CA dosage, CA extravasation kinetics, and DCE-MRI pulse sequence parameters (38). With the standard CA dose and a DCE-MRI sequence optimized for better SNR, that is also inherently water exchange sensitive (38), such as the case in this study, the most important factor that drives the DCE data sensitivity to k_io_ is tissue-specific CA extravasation. It has been shown that when a DCE-MRI time-course is insensitive to water exchange and k_io_ is still fitted as a variable, the returned k_io_ parameter often hits the FXL-limit fitting boundaries (39). In this study, the fitting upper boundary for k_io_ was set at 1,000 s^−1^ to minimize the occurrence of fitting procedures stopping at a parameter boundary prematurely. Because the fitting sensitivity of k_io_ for each voxel-based DCE time-course within the tumor ROIs strongly depends on voxel-based CA extravasation, which was unknown before PK modeling, voxel-based SSM modeling was initially performed for all voxel data within the tumor ROIs. These fitted k_io_ values were then filtered with a biologically meaningful and DCE-MRI achievable range of 0.1 s^−1^–20 s^−1^. A k_io_ of 0.1 s^−1^ or lower reflects that its reciprocal, the mean intracellular lifetime, is on the order of 10 s or larger. This is an unrealistically large value for relatively small sizes of breast tissue cells. For example, based on a spherical cell model, Sehy et al. estimated that for an intracellular water lifetime of 10 s, the “cell size” is on the order of ~300 μm (40), at least an order of magnitude higher than that of breast cancer cells (41). For the upper limit, a k_io_ value of 20 s^−1^ or higher indicates that the transmembrane water molecule exchange process represented by k (= k_io_ + k_oi_, where k_oi_ is the rate constant defining the process of water molecules entering the intracellular space from EES (42)) is even greater. An *in vivo* system with k >20 s^−1^ will appear to be closer to the FXL than the FXR condition in a breast DCE experiment with a single-dose CA administered intravenously. After this simple voxel-based k_io_ filtering, the fraction of tumor voxels with k_io_ within the range of 0.1 s^−1^–20 s^−1^ was recorded, and descriptive statistics were then used to summarize the filtered k_io_ results.

### Reporting of MRI metrics

For each patient at each MRI visit, the tumor LD, SER, and quantitative parameters from PK modeling were reported. For voxel-based analysis (SER and PK parameters), the mean tumor parameter value was calculated by averaging voxel parameter values. In addition, the median and width of the interquartile range (iqr = 75 percentile voxel parameter value − 25 percentile voxel parameter value) from the histogram analysis of the voxel parameter distribution were also obtained. For ROI-based analysis (PK parameters only), the derived parameter values from PK modeling of the single DCE time course were reported as the mean tumor parameter values. If multiple tumors were present, the average of the parameter value from each tumor was reported.

### Statistical data analysis

Descriptive statistical analysis was performed for each MRI metric at each visit, as well as the percent change relative to baseline (V1), such as V21% (percent change at V2 relative to V1) and V31% (percent change at V3 relative to V1), for each response group (pCR and non-pCR). Differences between the groups were assessed using the Wilcoxon rank-sum test. Student’s t-test was used to evaluate the differences in K^trans^ among the four PK analysis approaches of ROI- and voxel-based analysis in combination with *f*R_1,0_ and *m*R_1,0_, and between TM and SSM. Statistical significance was set at P-values <0.05.

In this preliminary study, the discriminative performances of V21% and V31% of each MRI metric for early prediction of pCR *vs.* non-pCR were evaluated using univariate logistic regression with ROC curves, and AUC values were calculated with 95% confidence intervals (CIs). All statistical analyses were performed using R: A Language and Environment for Statistical Computing (43).

## Results

### ROI- and voxel-based PK analysis with *f*R_1,0_ and *m*R_1,0_

**Figure 1:**
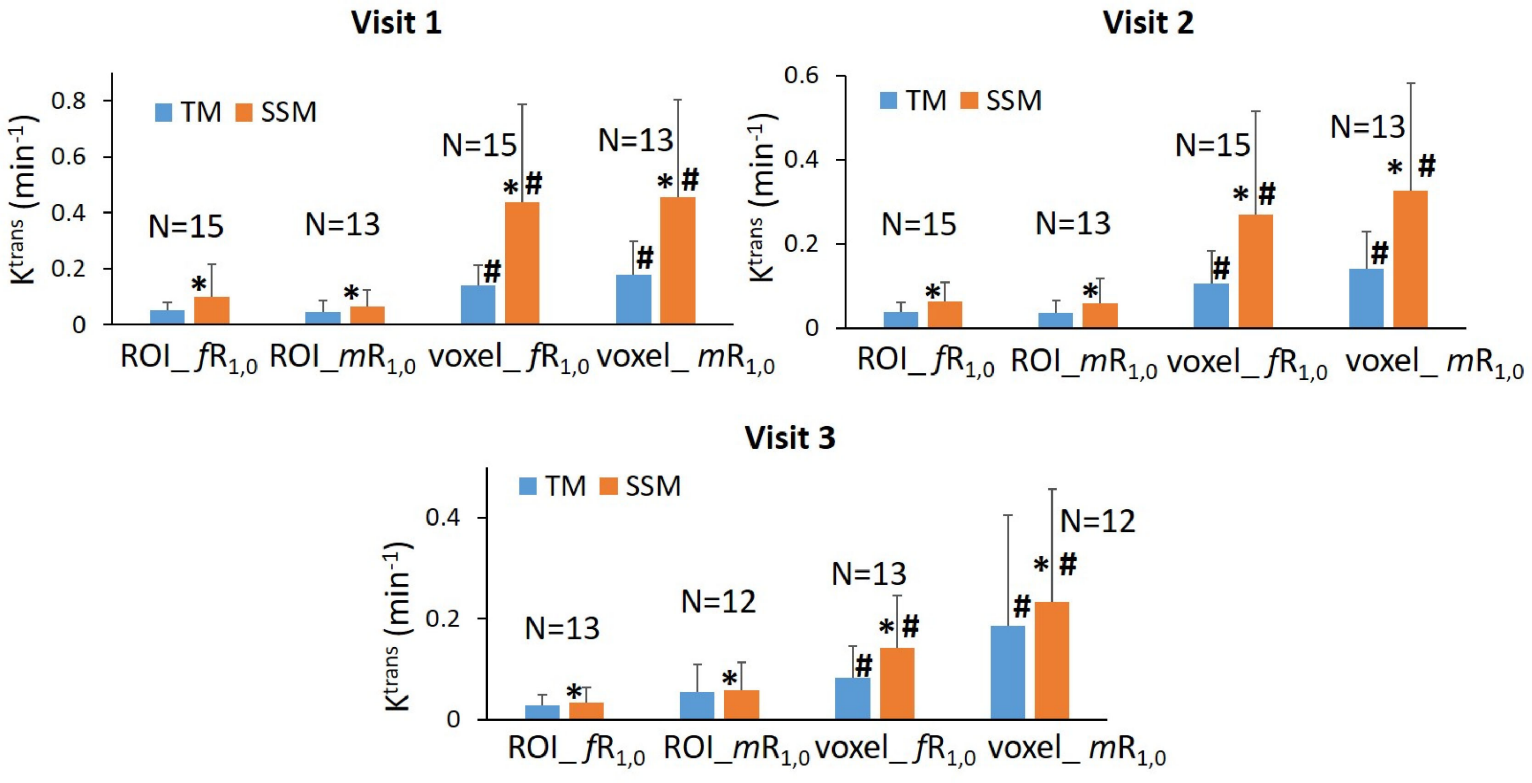
Column graphs of mean TM (blue) and SSM (orange) K^trans^ of the patient cohort under the analysis conditions of ROI_ *f*R_1,0_, ROI_*m*R_1,0_, voxel_ *f*R_1,0_, and voxel_ *m*R_1,0_ from Visit 1 (V1) to Visit 3 (V3). Error bars represent the positive standard deviation (SD). N: patient number; *statistically significant (P <0.05, t-tests) difference in K^trans^ between TM and SSM under the same analysis conditions; ^#^statistically significant (P <0.05, t-tests) difference in K^trans^ between ROI- and voxel-based analyses under the same R_1,0_ and PK model conditions.

**Figure 2:**
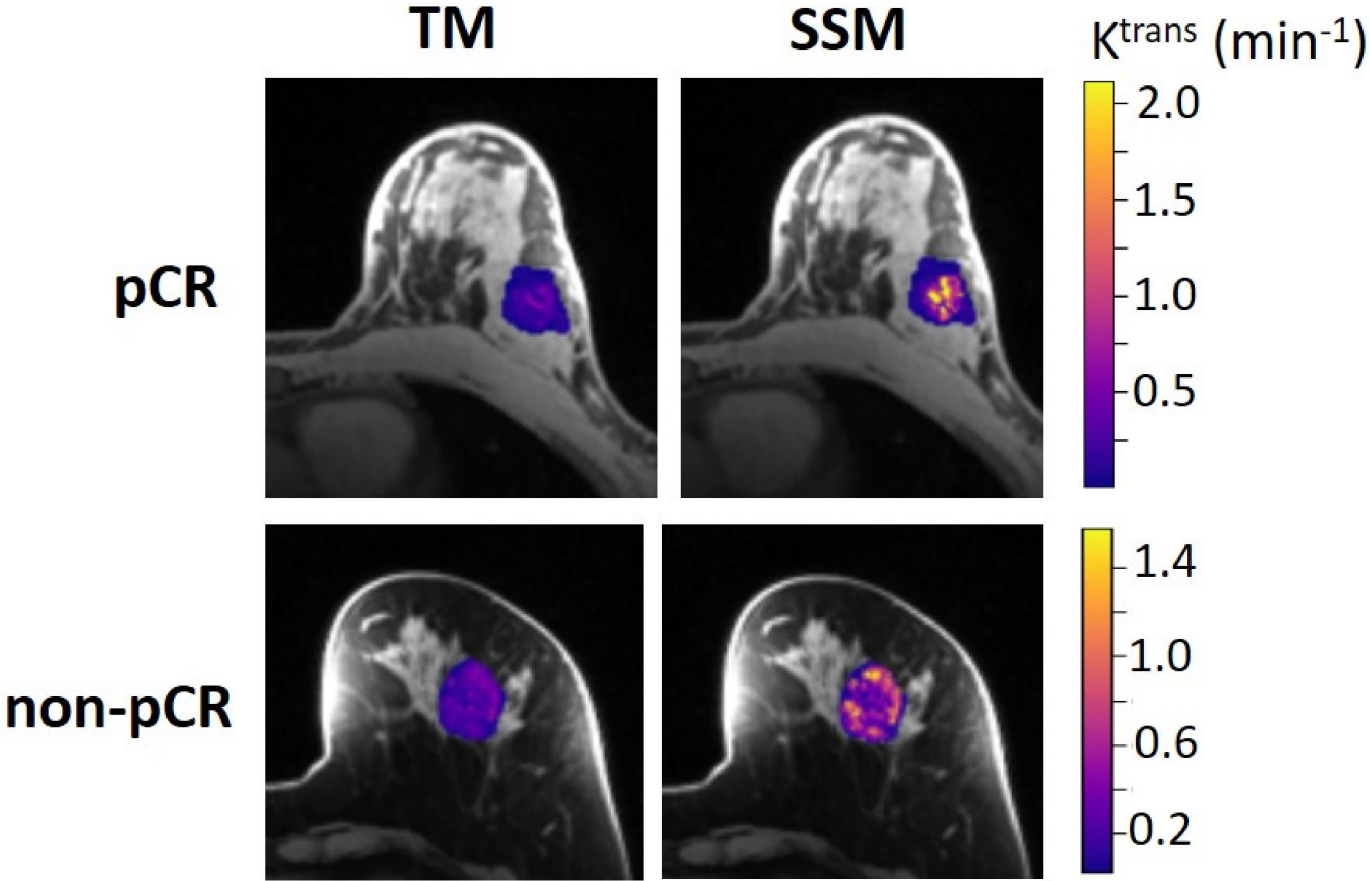
Voxel-based tumor K^trans^ color parametric maps for 43-year old pCR (left breast cancer, top row) and 53-year old non-pCR (left breast cancer, bottom row) patients at V1, obtained from TM (left column) and SSM (right column) PK analysis, respectively. For each patient, the K^trans^ maps (overlaid on post-contrast DCE images) from the same slice are shown, and the color scale is kept the same to allow the comparison of TM and SSM K^trans^. It can be clearly observed that SSM K^trans^ was substantially greater than TM K^trans^ in both tumors.

The results of the tumor mean K^trans^ from V1 to V3 (two patients missed V3 scans) and its performance for early prediction of NAC response are reported here. **Figure 1** shows column graphs of TM and SSM mean ± SD K^trans^ of the patient cohort under the analysis conditions of ROI_ *f*R_1,0_, ROI_*m*R_1,0_, voxel_ *f*R_1,0_, and voxel_ *m*R_1,0_. The VFA-measured phantom R_1_ and *in vivo* breast tumor R_1,0_ values from the two vendor platforms were in excellent agreement with the ground truth R_1_ of the fibroglandular tissue mimicking material and literature reported breast tumor R_1,0_ at 3T (25–27), respectively. However, due to technical reasons still under investigation, the corresponding VFA-measured R_1_ and R_1,0_ values from the other vendor platforms were overestimated by ≥100%, resulting in failure in PK modeling of patient DCE data from that platform. Thus, patient data from that platform (N = 2) were not included under the *m*R_1,0_ condition in **Figure 1**. SSM K^trans^ was significantly (P <0.05) larger than TM K^trans^ under all conditions, while K^trans^ from the voxel-based analysis was significantly (P <0.05) larger than that from the ROI-based analysis. There was no statistically significant difference in K^trans^ between *f*R_1,0_ and *m*R_1,0_ for either ROI- or voxel-based PK analysis using TM or SSM. **Figure 2** shows voxel-based V1 K^trans^ parametric maps of pCR and non-pCR tumors obtained from TM and SSM PK analyses using *f*R_1,0_. For each patient, color K^trans^ maps from the same image slice are shown for comparison of the TM and SSM analyses. It can be clearly observed that SSM K^trans^ was substantially greater than TM K^trans^ in both tumors.

**Table 3** lists the ROC AUC values of K^trans^ percent changes, V21% and V31%, for the early prediction of pCR (N = 6) *vs.* non-pCR (N = 9). SSM K^trans^ from voxel-based analysis exhibited a better predictive performance than TM K^trans^, with SSM K^trans^ under the condition of voxel_*f*R_1,0_ showing the highest predictive accuracy. Overall, for both TM and SSM K^trans^, voxel-based PK analysis using *f*R_1,0_ was the optimal approach for early prediction of NAC response at both V2 and V3. Additionally, the use of *f*R_1,0_ for PK analysis also allowed for inclusion of patient data from the vendor platform that produced substantial errors in VFA R_1_ measurement, which would otherwise be discarded if *m*R_1,0_ was used for PK analysis. Therefore, we proceeded to compare the quantitative DCE-MRI parameters derived with the voxel_*f*R_1,0_ approach with SER and tumor LD for early prediction of NAC response.

**Table 3:** Early prediction of breast cancer response to neoadjuvant chemotherapy using K^trans^ percent change under different analysis conditions.

PK Model	ROC AUC (95% CI)			
	ROI_*f*R_1,0_	ROI_*m*R_1,0_	voxel_*f*R_1,0_	voxel_*m*R_1,0_
V21% K^trans^				
TM	0.65 (0.35, 0.94)	0.71 (0.39, 1.0)	0.70 (0.42, 0.99)	0.67 (0.37, 0.96)
SSM	0.62 (0.39, 0.91)	0.70 (0.39, 1.0)	0.83 (0.62, 1.0)	0.78 (0.53, 1.0)
V31% K^trans^				
TM	0.88 (0.68, 1.0)	0.77 (0.44, 1.0)	0.93 (0.79, 1.0)	0.86 (0.64, 1.0)
SSM	0.82 (0.54, 1.0)	0.72 (0.39, 1.0)	0.98 (0.91, 1.0)	0.95 (0.84, 1.0)

ROC, receiver operating characteristic; AUC, area under the curve; CI, confidence interval; PK, pharmacokinetic; *f*R_1,0_, fixed R_1,0_; *m*R_1,0_, measured R_1,0_; V21%, MRI visit 2 (V2) relative to visit 1 (V1) percent change; V31%, MRI visit 3 (V3) relative to visit 1 (V1) percent change; TM, Tofts model; SSM, Shutter-Speed model.

### Early prediction of breast cancer response to NAC

**Figure 3:**
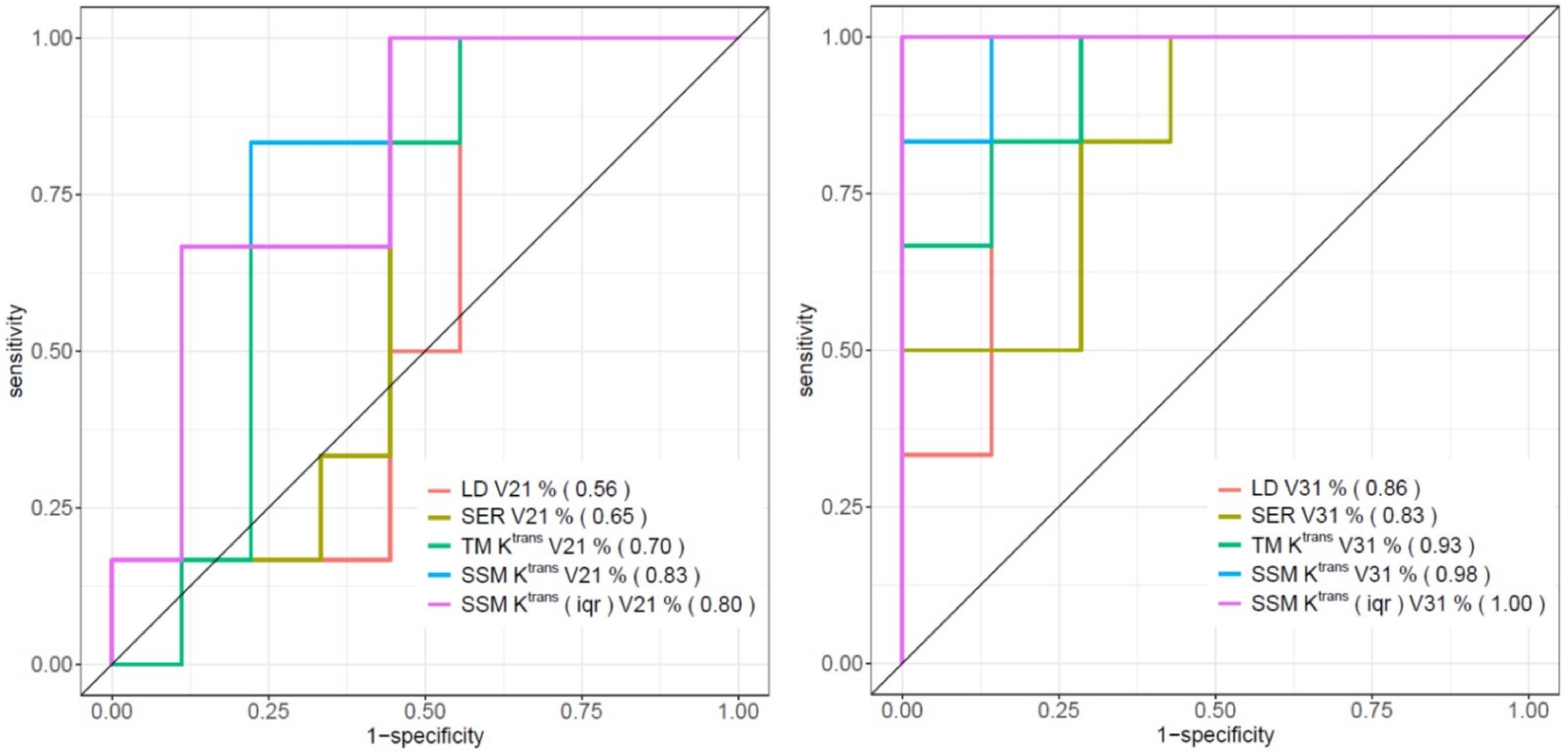
ROC curves of V21% (left) and V31% (right) of LD, SER (mean), TM K^trans^ (mean), SSM K^trans^ (mean), and SSM K^trans^ (iqr) for the early discrimination of pCR and non-pCR. AUC values are shown in parentheses in the figure legends.

**Figure 4:**
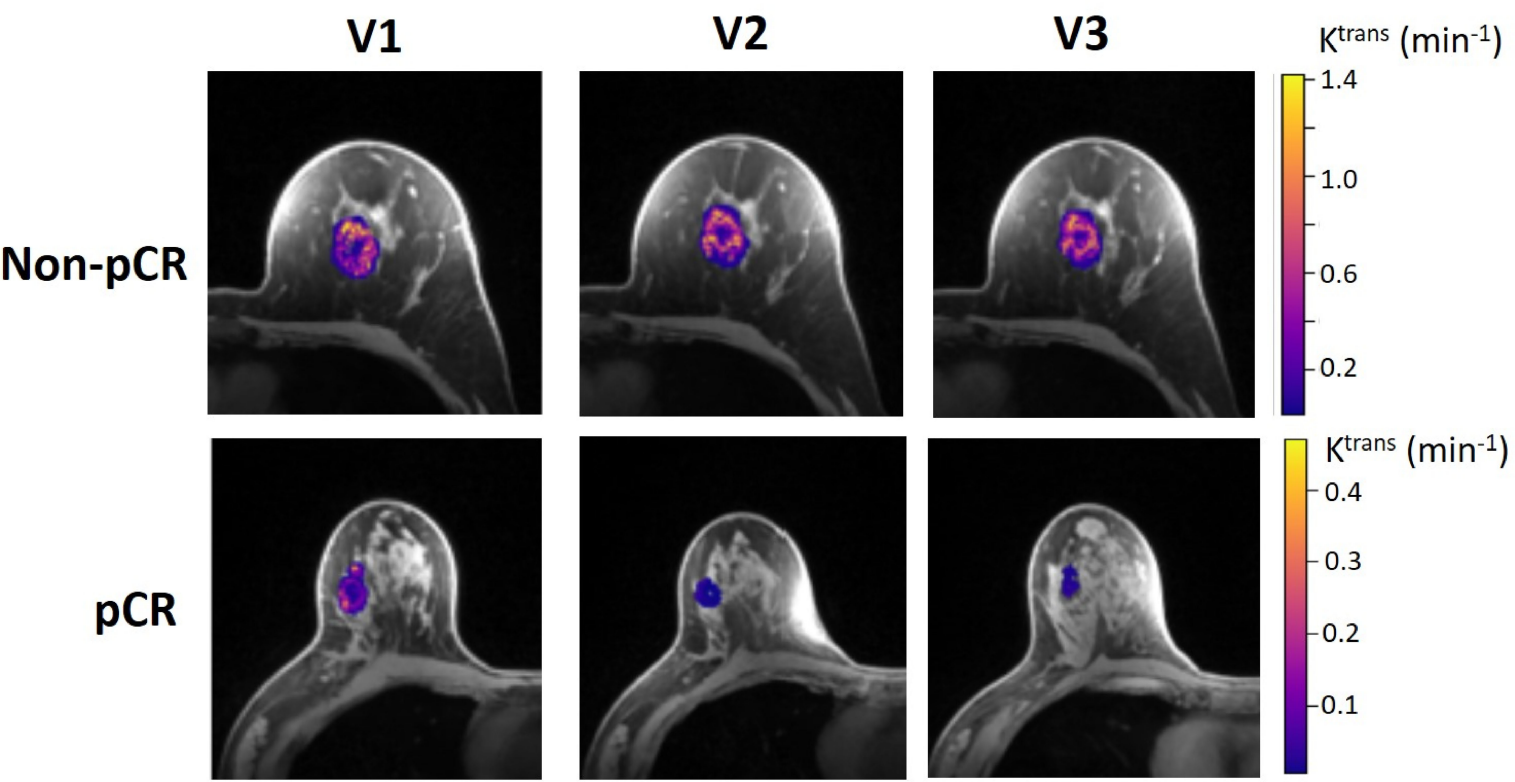
Voxel-based tumor SSM K^trans^ parametric maps in color (overlaid on a cropped post-contrast DCE image slice through the center of the tumor) at V1, V2, and V3 from a 52-year old non-pCR patient with left breast cancer (top) and 57-year old pCR patient with right breast cancer (bottom). For each tumor, the K^trans^ color scale was kept the same from V1 to V3 to allow the visual assessment of K^trans^ changes.

**Figure 5:**
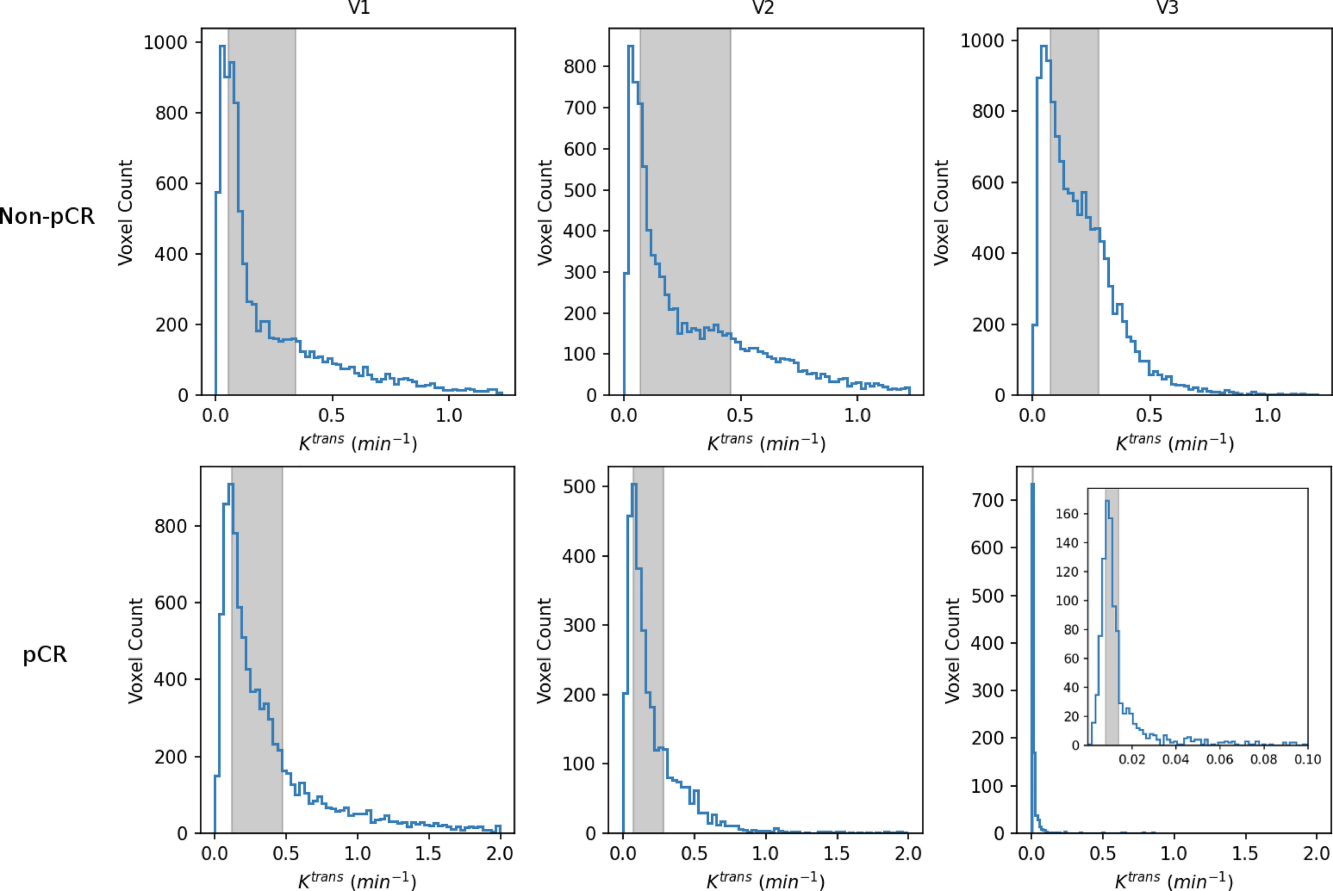
V1–V3 histograms of voxel SSM K^trans^ within the tumor ROIs from a non-pCR patient (top; the same patient as shown in **Figure 4**) and a 42-year old pCR patient with right breast cancer (bottom). The width of the gray column in each panel represents the iqr value. For each patient, the x-axis (K^trans^) scale was kept the same from V1 to V3 to demonstrate longitudinal changes in K^trans^ iqr. For the V3 histogram of the pCR patient, an inset with a much smaller K^trans^ scale (0 min^−1^–0.1 min^−1^) is shown for better visualization of this histogram.

**Table 4** shows the percent changes in MRI metrics (V21% and V31%) of the pCR and non-pCR groups, P-values from the Wilcoxon test comparing the two groups, and ROC AUC values for the early prediction of pCR *vs.* non-pCR. For the semi-quantitative SER parameter, percent changes in tumor mean SER showed higher AUC values than those of median SER and are listed in **Table 4**. The results for the PK parameters reported here were all obtained using the voxel_*f*R_1,0_ approach, and only those with percent changes showing AUC ≥0.80, indicating good predictive performance, are summarized in **Table 4**. After the first NAC cycle, V21% of LD was a poor predictor of response with AUC = 0.56, whereas V21% of SER and several SSM PK parameters showed fair to good predictive performance with SSM K^trans^ (mean), v_e_ (mean), K^trans^ (iqr), and k_ep_ (iqr) showing AUC values of 0.83, 0.81, 0.80, and 0.81, respectively. None of the TM parameters demonstrated an AUC ≥0.80 after the first NAC cycle. At NAC midpoint, while V31% of both LD and SER demonstrated similar good predictive performances with AUC = 0.86 and 0.83, respectively, V31% of TM and SSM K^trans^ and k_ep_, whether the mean or median value, were excellent predictors with AUC >0.90. Furthermore, both SSM K^trans^ (iqr) and k_ep_ (iqr) percent changes separated the two groups completely, with AUC = 1. The associated, representative ROC curves are shown in **Figure 3**. **Figure 4** shows the tumor SSM K^trans^ parametric color maps at V1, V2, and V3 for a non-pCR and pCR patient. There were no noticeable changes in K^trans^ of the non-pCR tumor from V1 to V3. However, the decrease in K^trans^ was substantial in the pCR tumor from V1 to V2, and the values remained low at V3. **Figure 5** shows histograms of voxel SSM K^trans^ values for a pCR and a non-pCR tumor, from V1 to V3. The K^trans^ iqr values indicated by widths of the grey columns were 0.33 min^−1^, 0.44 min^−1^, and 0.21 min^−1^ for the non-pCR and 0.42 min^−1^, 0.21 min^−1^, and 0.0072 min^−1^ for the pCR, respectively, from V1 to V3. The difference in iqr changes at V2 and V3 relative to V1 is striking between these two tumors: a 33% increase at V2 and 36% decrease at V3 for the non-pCR, while a 50% decrease at V2 and 98% decrease at V3 for the pCR group.

**Table 4:** Early prediction of breast cancer response to neoadjuvant chemotherapy.

% Change of MRI Metric	Non-pCR (N = 9)Median (IQR)	pCR (N = 6)Median (IQR)	Wilcoxon P-Value	ROC AUC(95% CI)
V21%				
LD	−9 (−22, 0)	−9 (−12, −7)	0.80	0.56 (0.24, 0.87)
SER (mean)	0 (−19, 2)	−11 (−17, −8)	0.40	0.65 (0.34, 0.96)
SSM K^trans^ (mean)	1 (−55, 55)	−71 (−72, −59)	0.036	0.83 (0.62, 1.0)
SSM v_e_ (mean)	0 (−9, 2)	8 (6, 11)	0.050	0.81 (0.57, 1.0)
SSM K^trans^ (iqr)	−40 (−61, 6)	−78 (−83, −61)	0.066	0.80 (0.55, 1.0)
SSM k_ep_ (iqr)	−48 (−51, 27)	−64 (−71, −57)	0.050	0.81 (0.58, 1.0)
V31%				
LD	−33 (−43, −16)	−60 (−91, −51)	0.038	0.86 (0.64, 1.0)
SER (mean)	−4 (−30, 3)	−43 (−49, −33)	0.051	0.83 (0.60, 1.0)
TM K^trans^ (mean)	−17 (-29, 13)	−83(−88, −74)	0.0080	0.93 (0.79, 1.0)
TM K^trans^ (median)	−21 (−32, −7)	−82 (−86, −70)	0.0050	0.95 (0.84, 1.0)
SSM K^trans^ (mean)	−27 (−55, −5)	−91 (−95, −86)	0.0020	0.98 (0.91, 1.0)
SSM K^trans^(median)	−23 (−48, 6)	−88 (−90, −84)	0.0020	0.98 (0.91, 1.0)
TM k_ep_ (mean)	−1 (−12, 30)	−81 (−89, −74)	0.0050	0.95 (0.84, 1.0)
TM k_ep_ (median)	−17 (-25, 14)	−82 (−92, −71)	0.0050	0.95 (0.84, 1.0)
SSM k_ep_ (mean)	−27 (−39, −8)	−88 (−94, −82)	0.0080	0.93 (0.78, 1.0)
SSM k_ep_ (median)	−24 (−34, 38)	−91 (−94, −83)	0.0020	0.98 (0.91, 1.0)
SSM K^trans^ (iqr)	−62 (−65, −33)	−96 (−97, −93)	0.0010	1.0 (1.0, 1.0)
TM k_ep_ (iqr)	−15 (−23, −5)	−80 (−87, −65)	0.0080	0.93 (0.78, 1.0)
SSM k_ep_ (iqr)	−39 (−45, −21)	−93 (−95, −91)	0.0010	1.0 (1.0, 1.0)

V21%, MRI visit 2 (V2) relative to visit 1 (V1) percent change; V31%, MRI visit 3 (V3) relative to visit 1 (V1) percent change; LD, longest diameter; SER, signal enhancement ratio; TM, Tofts model; SSM, Shutter-Speed model; IQR, interquartile range; iqr, width of interquartile range = 75 percentile voxel parameter value—25 percentile voxel parameter value; ROC, receiver operating characteristic; AUC, area under the curve; CI, confidence interval.

### Advanced processing involving k_io_ filtering in SSM analysis

**Figure 6:**
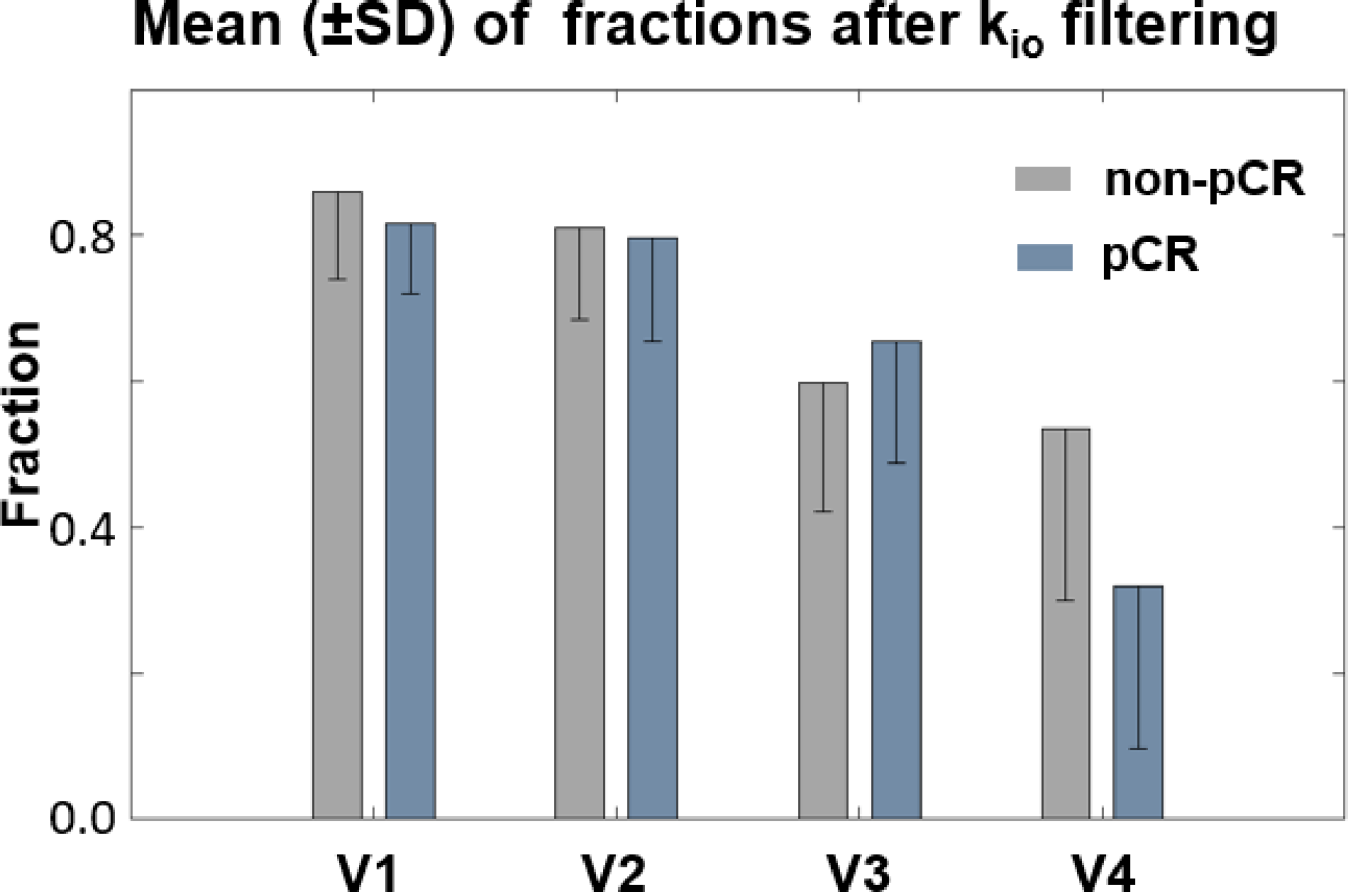
Fraction means (SD error bars) of the filtered k_io_ within the tumor ROIs for the two response groups were plotted. The fraction of voxels with filtered k_io_ values generally decreased from V1 to V4. The difference between V1 and V4 was highly substantial for both the non-pCR (gray) and pCR (blue) groups. Furthermore, the V4 fraction of filtered k_io_ was much smaller for pCRs than for non-pCRs.

**Figure 6** summarizes the fraction means (SD error bars) of the filtered k_io_ within the tumor ROIs for the two response groups. The fractions of voxels with filtered, meaningful k_io_ generally decreased from V1 to V4. The difference between V1 and V4 was substantial for both the non-pCR (gray) and pCR (blue) groups. Furthermore, the V4 fractions of pCRs were much smaller than those of non-pCRs. In addition, fraction means showed little R_1,0_-selection dependence between the use of *f*R_1,0_ and *m*R_1,0_ (data not shown). Both V21% and V31% of filtered k_io_ mean or median had ROC AUC values <0.80 for early prediction of NAC response.

**Figure 7:**
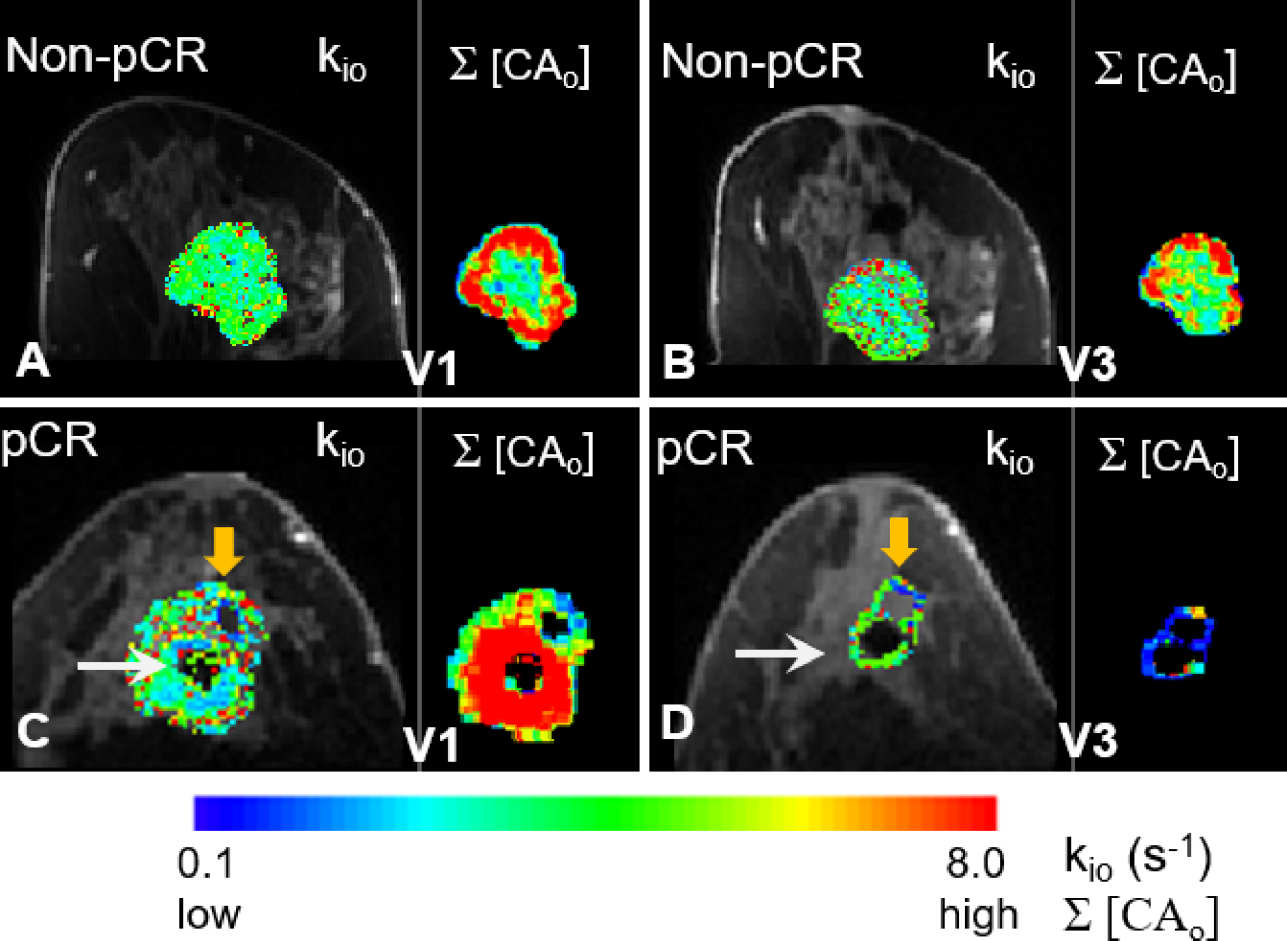
Filtered tumor ROI k_io_ results at two visits (V1 and V3) for a non-pCR **(A, B)** and a pCR **(C, D)** patient are shown. In each panel, k_io_ color map overlaid on a zoomed post-contrast DCE image is shown on the left, and the Σ[CA_o_] map on the right shows the voxel-based summation of the EES CA concentration, [CA_o_], over the entire DCE time course within the tumor ROI. In all four panels, results from the center slice of the respective tumors are shown. The white arrows in **(C, D)** point to the artifacts caused by a metal biopsy clip. A larger area of the pCR tumor was filtered out in the V3 k_io_ map [**(D)**, orange arrow] compared to a smaller filtered-out area in the V1 k_io_ map [**(C)**, orange arrow]. There were no noticeable filtered k_io_ areas in the non-pCR tumor at either V1 or V3.

In **Figure 7**, filtered tumor ROI k_io_ results at two visits (V1, V3) for a non-pCR (A, B) and a pCR (C, D) patient are shown. In each panel, the filtered k_io_ color map overlaid on a zoomed post-contrast DCE image is shown on the left, and the voxel-based Σ[CA_o_] map on the right shows the summation of the EES CA concentration, [CA_o_], over the entire DCE time-course within the tumor ROI. In all four panels, the results from the center slice of the respective tumors are shown. The white arrows in (C) and (D) indicate artifacts caused by a metal biopsy clip. In the pCR tumor, a larger area of k_io_ was filtered out at V3 (D, orange arrow) compared to a smaller filtered-out area at V1 (**C**, orange arrow) due to k_io_ filtering, in addition to our built-in quality control that masked out unenhanced voxels. Σ[CA_o_] approximates the total CA extravasation during the entire DCE acquisition and serves as a simple quantitative surrogate for monitoring the sensitivity of the voxel DCE time-course to water exchange kinetics. Cold spots in the Σ[CA_o_] maps, where CA extravasation was low, matched the areas of the filtered-out k_io_ quite well, supporting the validity of our filtering approach to a certain degree.

## Discussion

To the best of our knowledge, this is the first MC and MP study using quantitative DCE-MRI to predict breast cancer response to NAC, where GRE-based product sequences of k-space undersampling during acquisition and view-sharing during reconstruction from Siemens, Philips, and GE platforms were used for high spatial and temporal resolution breast DCE-MRI. The data acquisition scheme used for DCE-MRI in this MC and MP setting allows for bilateral full breast coverage with adequate spatial resolution for accurate morphological evaluation, as well as sufficient temporal resolution for quantitative PK analysis of the breast DCE time-course data (14, 15). In today’s SoC breast DCE-MRI protocols, owing to trade-offs between spatial and temporal resolution in conventional sequences, the necessity for accurate tumor morphology assessment with high spatial resolution and spatial coverage results in low temporal resolutions between 60 s and 120 s, which precludes meaningful PK modeling of time-course data with acceptable accuracy (15, 16). With potentially faster dynamic imaging methods available from vendors through combinations of accelerated data acquisition approaches and advanced reconstruction algorithms (15), the ability to acquire breast DCE-MRI with simultaneous high spatial and temporal resolutions using vendor product sequences may facilitate the translation of quantitative DCE-MRI into clinical workflow.

With inherent differences in hardware and software among the three major MRI vendor platforms, standardizations in VFA and DCE acquisition parameters were implemented across the three sites to minimize differences in the acquired data and consequently, variations in results from data analysis (14, 15, 17). These included the use of the same three FAs in VFA acquisition for R_1,0_ mapping, and the same center and peripheral portions of the k-space in the three vendor-specific k-space undersampling and view-sharing product sequences for DCE-MRI acquisition. In addition, the same FA (10°), minimal TE (0.9 ms–2.9 ms), and similar TR in the range of 5.0 ms–6.2 ms were used across the platforms to ensure DCE data sensitivity to the water exchange effects (38), supporting the use of the SSM for PK analysis. Despite efforts in data acquisition standardization and centralized data analysis using a single software tool for both VFA R_1_ fitting and DCE-MRI PK modeling, VFA measurements of phantom R_1_ and patient tumor R_1,0_ on one vendor platform resulted in substantial biases compared to ground truth values in the phantom and measurements on the other two platforms, as well as literature reported breast tumor R_1,0_ values (25–27). The ≥100% overestimation of tumor R_1,0_ on this platform also caused patient data fitting failures when using either the TM or SSM. Therefore, PK modeling of patient data from this vendor platform was performed using *f*R_1,0_ only, excluding the use of *m*R_1,0_ from the analysis. The sources of errors in VFA R_1_ mapping on this platform are still under investigation. VFA fitting is inherently challenging owing to the need to fit multiple variables, which requires numerous individual images of unique FAs. Because this is not practical owing to the scan time, several assumptions are typically made to simplify the fitting, including the linearity and spatial uniformity of B_1_ over multiple transmitted power settings, as well as the direct correspondence between the delivered FA of the independent B_1_ mapping sequence and the VFA sequence. These assumptions rely on the use of non-clinically validated research tools from vendor platforms. However, these assumptions may not always hold true on specific vendor implementations. Furthermore, each vendor uses a different method to map the FA. There are no standards for the reporting of FA or B_1_ maps, including units, presenting challenges when assimilating data across multivendor platforms. One valuable lesson learned here for implementing MC and MP quantitative DCE-MRI is the importance of QA/QC scans of phantoms with ground truth R_1_ values to determine whether all vendor platforms provide reliable R_1_ measurements. If this is not the case, either error sources should be identified and corrective actions taken, or an alternative solution, such as the use of *f*R_1,0_ for PK analysis, should be found.

In this study, using K^trans^ as the reference imaging biomarker for the prediction of breast cancer response to NAC, we found that voxel-based analysis using a literature-reported *f*R_1,0_ value was the optimal approach for PK analysis of DCE-MRI data collected from the MC and MP settings, whether the TM or SSM was used for data modeling. This approach allowed the inclusion of data from one platform, where the *m*R_1,0_ values calculated from VFA measurements were unreliable. The statistically insignificant differences in K^trans^ between the use of *f*R_1,0_ and *m*R_1,0_ (**Figure 1**) suggest that it is reasonable and practical to use *f*R_1,0_ for PK modeling (44), which can mitigate random and systematic errors from R_1,0_ measurement across vendor platforms and potentially eliminate the need for B_1_ mapping and VFA acquisition in MC and MP trials. Although the use of *f*R_1,0_ instead of *m*R_1,0_ in PK analysis is expected to cause systematic errors in the estimated PK parameters, unlike random errors, the impact of systematic errors is lessened when percent changes in PK parameters, such as V21% and V31%, are used in a longitudinal study to predict breast cancer response to NAC. Compared with voxel-based analysis, ROI-based analysis dilutes tumor heterogeneity in perfusion and permeability, resulting in significantly smaller K^trans^ values and a narrower range of K^trans^ changes in response to therapy. The latter may potentially reduce the predictive performance of K^trans^ for NAC response. Another disadvantage of ROI-based analysis is the inability to assess changes in PK parameter heterogeneity in response to treatment.

The preliminary results from this MC and MP study show that after only one NAC cycle, semi-quantitative and quantitative DCE-MRI metrics outperformed tumor size measurement in the early prediction of breast cancer response to NAC. The quantitative parameter K^trans^ consistently provided a more accurate prediction of NAC response than both size measurement (LD) and SER after the first NAC cycle and at the NAC midpoint. These findings agree with many similar studies that used DCE-MRI to assess the breast cancer response to NAC (12, 13). The larger decreases in K^trans^ (iqr) and k_ep_ (iqr) in pCRs compared to non-pCRs at V2 and especially V3 indicate greater decreases in tumor perfusion/permeability heterogeneity in patients responding to NAC regimens.

Consistent with previous studies (36, 45), SSM K^trans^ was substantially greater than TM K^trans^ in this cohort of malignant breast tumors when DCE-MRI acquisition was sensitive to the water-exchange effect (38). Since there is no significant difference between SSM and TM K^trans^ in benign breast lesions or normal tissue (36, 45), SSM K^trans^ potentially has better predictive performance than TM K^trans^ due to the former’s greater dynamic ranges of change in response to therapy, assuming that microvascular properties of a responding tumor shift towards those of a benign lesion or normal tissue. This is manifested by the fact that, after the first NAC cycle, while V21% of mean SSM K^trans^ was a good predictor of NAC response with an ROC AUC value of 0.83, V21% of mean TM K^trans^ was only a fair predictor with ROC AUC = 0.70 (not shown in **Table 4**). Although at the NAC midpoint, both SSM and TM V31% K^trans^ (and k_ep_) are excellent predictors of NAC response, the percent changes of SSM K^trans^ and k_ep_ were larger than those of the TM counterparts, and the P-values from comparing the two response groups were generally smaller for the SSM parameters.

Our initial experience shows that when the water exchange effect is explicitly modeled in the SSM analysis of DCE-MRI data, the k_io_ parameter may provide complementary information to the more commonly modeled K^trans^ parameter, which only focuses on CA kinetics. Recent studies have shown that k_io_ is an imaging biomarker of metabolic activity (46). In fact, V31% of the tumor mean k_io_ was a fair early predictor of NAC response with an ROC AUC value of 0.71 (not shown in **Table 4**). Combining K^trans^ and k_io_ in a multivariate predictive model may further improve the predictive accuracy for NAC response. NAC regimens often reduce tumor permeability/vascularity (21), resulting in reduced interstitium [CA] during a DCE study. This, in turn, makes k_io_ quantification less reliable. In other words, reduced CA extravasation results in a smaller R_1_ difference between extracellular and intracellular spaces. This decreases the DCE-MRI sensitivity to the water exchange effect and, consequently, negatively impacts the accuracy and precision of SSM quantification of k_io_. Therefore, caution should be exercised when evaluating the estimated k_io_ values from the SSM analysis. Unreliable voxel k_io_ values should be filtered out. The smaller fraction of filtered k_io_ at V4 in pCRs compared to non-pCRs quantitatively reflected lower CA extravasation in the former, consistent with the K^trans^ and k_ep_ results. The right side of each panel in **Figure 7** represents a simple method for quantitatively estimating the extent of CA extravasation using Σ[CA_o_]. As expected, the pCR tumor at V3 showed low Σ[CA_o_] owing to reduced perfusion and permeability in response to NAC treatment, resulting in large areas of unreliable k_io_ being filtered out. A more accurate measure of voxel DCE data sensitivity to water exchange should include an estimation of the extent and duration of |R_1i_ − R_1o_| absolute difference between intracellular and extracellular R_1_ exceeding (or at least close to) the exchange kinetics defined by k, the transmembrane water molecule exchange process (42). However, this may add complications in translational studies, such as this MC and MP study.

Our model-based approach for estimating BAT eliminated the need to manually align the AIF with voxel-based tissue DCE curves in PK analysis, which improves automation in the entire data processing workflow. Manual inspection of the model-selected BATs showed robust and qualitatively good performance. Future work will include quantifying the performance using a digital phantom for comparison with manual alignment.

There are several limitations to this preliminary MC and MP study. The main limitation is the small sample size of 15 patients in total, which caused large 95% CI ranges of the ROC AUC values for the prediction of NAC response and may artificially inflate the predictive performances of the quantitative PK parameters. The small sample size at each site also renders cross-vendor platform comparison of results unreliable, and therefore, was not performed. Second, for correlation analysis between MRI metrics and pathologic response outcomes, the small sample size precluded meaningful analysis stratified by breast cancer subtypes, as shown in **Table 1**. Therefore, the results presented here are more reflective of those from the general breast cancer population treated with SoC NAC regimens. Third, we did not perform a multivariate analysis by combining clinicopathological features with individual MRI metrics with high predictive performance, which may further enhance the capability of early prediction of NAC response, especially after the first NAC cycle. Fourth, we only used the iqr of voxel-based parameters to characterize changes in tumor heterogeneity in response to NAC, without performing a more comprehensive radiomics analysis (47), which may provide better predictive performance. Lastly, only three FAs within a relatively narrow range were used in VFA measurements of phantom R_1_ and breast tumor R_1,0_. In clinical practice, where breast tumor R_1,0_ values are unknown and could vary greatly, using more FAs over a larger range may result in more accurate R_1,0_ mapping, and consequently, more accurately estimated PK parameters. However, the usual time constraint for a clinical MRI protocol makes it difficult to add more FAs to the VFA acquisition. Furthermore, the small number of FAs used in this study is unlikely to be the reason why one vendor platform returned substantially biased R_1_ and R_1,0_ values.

In conclusion, the initial results from this MC and MP study validate findings from many single-site studies that quantitative DCE-MRI is superior to tumor size measurement for the prediction of breast cancer response to NAC. Both SSM and TM K^trans^ showed better predictive performance than the semi-quantitative SER metric. Furthermore, K^trans^ and k_ep_ derived from the SSM, using DCE-MRI data acquired with sensitivity to the water exchange effect, generally performed better than the TM counterparts in the prediction of NAC response, especially after only one cycle of NAC. Due to potential large variations in the accuracy of VFA-measured R_1,0_ on different vendor platforms, SSM PK analysis using a fixed, literature-reported breast tumor R_1,0_ could be a best-practice approach in quantitative DCE-MRI prediction of breast cancer response to NAC in an MC and MP setting.

## Data Availability

The raw data supporting the conclusions of this article will be made available by the authors, without undue reservation.
