# Supplementary material for: Initial experience in implementing quantitative DCE-MRI to predict breast cancer therapy response in a multi-center and multi-vendor platform setting
Source: Front Oncol. 2024 Nov 29;14:1395502. doi: 10.3389/fonc.2024.1395502 (PMC11638047; doi:10.3389/fonc.2024.1395502)
Supplement: Supplementary file 1 [file DataSheet1.docx]

Supplementary Material

**Initial Experience in Implementing Quantitative DCE-MRI to Predict Breast Cancer Therapy Response in a Multi-Center and Multi-Vendor Platform Setting**

Brendan Moloney, Xin Li*, Michael Hirano, Assim Saad Eddin, Jeong Youn Lim, Debosmita Biswas, Anum S. Kazerouni, Alina Tudorica, Isabella Li, Mary Lynn Bryant, Courtney Wille, Chelsea Pyle, Habib Rahbar, Su Kim Hsieh, Travis L. Rice-Stitt, Suzanne M. Dintzis, Amani Bashir, Evthokia Hobbs, Alexandra Zimmer, Jennifer M. Specht, Sneha Phadke, Nicole Fleege, James H. Holmes, Savannah C. Partridge, Wei Huang^*^

**DCE-MRI Pharmacokinetic Modeling**

The standard Kety-Schmidt type of pharmacokinetic (PK) rate law, equation (S1), was used for contrast agent (CA) kinetics quantification,

$C_{t}(t)=K^{\mathrm{trans}}\int_{0}^{t} C_{p}\left( t^{'} \right)\exp\left( -K^{\mathrm{trans}}/v_{e}\left( t-t^{'} \right) \right)dt^{'}$ (S1)

where C_t_(t) and C_p_(t) are the tissue and blood plasma CA concentrations ([CA]) at time t, respectively. The latter is often called arterial input function (AIF). A literature reported population-averaged AIF (1) measured from an axillary artery from a previous single-breast DCE study in the sagittal plane was adopted for all PK modeling in this study. K^trans^ is the CA volume transfer rate constant between blood plasma and extravascular, extracellular space (EES), and v_e_ is the EES volume fraction. The rate constant, k_ep_, is calculated as K^trans^/v_e_.

Since water proton signal (rather than signals from CA molecules directly) is detected in DCE-MRI and the presence of CA is quantified based on its effect in increasing the R_1_ relaxation rate constant of the water proton signal, voxel water proton R_1_ time-course was first derived using Eq. (2) in the main text. Unlike the VFA approach where the independent variable is α, R_1_ time-course was calculated from DCE baseline signal (with R_1_ = R_1,0_) and post CA injection DCE signals with the DCE frame imaging time as the independent variable. Using B_1_-corrected α values (from an accurate B_1_ map) is a critical and often neglected aspect in producing an accurate R_1_ time-course.

In addition to R_1_ quantification, the relationship of [CA] and proton R_1_ is part of the DCE-MRI PK modeling. For this, the most commonly assumed fast-exchange-limit (FXL) linear relationship, Eq (S2), as well as the simplest fast-exchange-regime (FXR) non-linear relationship, Eq. (S3), were used in this study. The FXL approach is often called the Tofts model (TM) (2, 3) and the FXR approach, which takes into account the finite trans-cell membrane water exchange kinetics, belongs to the Shutter-Speed models (SSM) (4, 5). Using the FXL approach:

R_1_ =R_1,0_ + r_1_·[CA] (S2)

where r_1_ (s^-1^mM^-1^) is the CA relaxivity, which describes the potency of the CA in accelerating the water proton T_1_ relaxation (∝ΔR_1_ = R_1_ - R_1,0_). Alternatively, using the FXR approach:

$R_{1}=\frac{1}{2}\left[ \left( R_{1i}+R_{1o} \right)+k \right]-\frac{1}{2}\left\{ \left[ \left( R_{1i}-R_{1o} \right)+\left( k_{io}-k_{oi} \right) \right]^{2}+4k_{io}k_{oi} \right\}^{1/2}$ (S3)

where the R_1i_ (“i” for inside) and R_1o_ (“o” for outside) are the intrinsic intracellular and extracellular water proton relaxation rate constants, respectively; k_io_ is the unidirectional cellular water efflux rate constant; k_oi_ is the rate constant defining the process of water molecules entering the intracellular space from the EES; and k = k_io_ +k_oi_ defines the transmembrane water molecule exchange process (6). The comparison of k with |R_1i_ - R_1o_| defines the system’s water exchange condition (5). The |R_1i_ - R_1o_| quantity varies during CA passage and an increased value of this quantity makes the exchange system appear to slow down. When [CA] in a DCE-MRI experiment is sufficiently large and |R_1i_ - R_1o_| >> k, the so-called slow exchange can be achieved and one can observe multiple R_1_ values. However, slow exchange is rarely achieved in *in vivo* DCE-MRI experiments (7), and a single-valued R_1_ with a nonlinear R_1_ vs. [CA] relationship is more likely to be observed. As an exchange-sensitized expression, Eq. (S3) provides a nonlinear relationship between R_1_ and [CA] when |R_1i_ - R_1o_| approaches k (FXR condition) and it reduces to Eq. (S2) when |R_1i_ - R_1o_| << k (FXL condition). For our DCE modeling approach, R_1i_ and R_1o_ are both assumed to be equal to R_1,0_ before CA arrival. During CA passage, R_1o_ varies according to Eq. (S2) while R_1i_ remains unchanged due to the fact that CA molecules generally do not enter the intracellular space. Under the FXL condition, R_1o_ and R_1i_ become indistinguishable and the linear relationship between a single tissue R_1_ value and [CA] (Eq. (S2)) holds. In summary, PK analysis of the DCE-MRI data in this study was performed with both the FXL (Eq. (S2)) and FXR (Eq. (S3)) models, denoted as the TM and SSM, respectively.

**References:**

1. Huang W, Tudorica LA, Li X, Thakur SB, Chen Y, Morris EA, et al. Discrimination of benign and malignant breast lesions by using shutter-speed dynamic contrast-enhanced MR imaging 1. Radiology. 2011;261(2):394-403.

2. Tofts PS, Brix G, Buckley DL, Evelhoch JL, Henderson E, Knopp MV, et al. Estimating kinetic parameters from dynamic contrast-enhanced T(1)-weighted MRI of a diffusable tracer: standardized quantities and symbols. Journal of magnetic resonance imaging : JMRI. 1999;10(3):223-32.

3. Tofts PS, Benton CE, Weil RS, Tozer DJ, Altmann DR, Jager HR, et al. Quantitative analysis of whole-tumor Gd enhancement histograms predicts malignant transformation in low-grade gliomas. Journal of magnetic resonance imaging : JMRI. 2007;25(1):208-14.

4. Yankeelov TE, Rooney WD, Li X, Springer Jr CS. Variation of the Relaxographic "Shutter-Speed" for Transcytolemmal Water Exchange Affects the CR Bolus-Tracking Curve Shape. Magnetic Resonance in Medicine. 2003;50(6):1151-69.

5. Li X, Rooney WD, Springer Jr CS. A unified magnetic resonance imaging pharmacokinetic theory: Intravascular and extracellular contrast reagents. Magnetic Resonance in Medicine. 2005;54(6):1351-9.

6. Li X, Mangia S, Lee JH, Bai R, Springer CS, Jr. NMR shutter-speed elucidates apparent population inversion of (1) H2 O signals due to active transmembrane water cycling. Magn Reson Med. 2019;82(1):411-24.

7. Springer CS, Jr. Using (1)H2O MR to measure and map sodium pump activity in vivo. J Magn Reson. 2018;291:110-26.
